# Barriers and Motives for Complying With “Sexual Distancing” Among Men Who Have Sex With Men During the First COVID-19 Pandemic Lockdown in Amsterdam: A Qualitative Study

**DOI:** 10.1097/OLQ.0000000000001636

**Published:** 2022-04-09

**Authors:** Dominique C. de Vries, Hanne M.L. Zimmermann, Susanne Drückler, Udi Davidovich, Elske Hoornenborg, Henry John C. de Vries

**Affiliations:** From the ∗ Department of Infectious Diseases, Public Health Service of Amsterdam; † Department of Social Psychology, University of Amsterdam; ‡ Amsterdam UMC, University of Amsterdam, Academic Medical Center, Department of Dermatology, Amsterdam Institute for Infection and Immunity (AIII), Amsterdam, the Netherlands

## Abstract

**Background:**

Between March 15 and May 31, 2020, the Dutch government imposed lockdown and health measures to curb the coronavirus disease 2019 (COVID-19) pandemic. As part of social distancing, sexual distancing was one of these measures. Sexual distancing implied refraining from sex with partners outside of one's household. We aimed to elucidate barriers and motives for complying with sexual distancing and related factors that could have led to (non)compliance among men who have sex with men.

**Methods:**

In this exploratory qualitative study, we interviewed men who have sex with men who visited the center for sexual health in Amsterdam during the first COVID-19 lockdown using a semistructured interview guide from March to May 2020. We interviewed both men who complied and did not comply with sexual distancing. The interviews were transcribed verbatim and analyzed using an open-coding process in MAXQDA.

**Results:**

We included 18 noncompliers and 4 compliers to COVID-19 sexual distancing. Barriers to compliance were the following: lack of information on, or understanding of, the need for sexual distancing; being single; having had an active sex life before COVID-19; a high perceived importance of the social aspect of sex; a strong urge for sex; using sex to cope with the negative impact of the pandemic; being under the influence of alcohol or drugs; and not perceiving COVID-19 as a serious health threat. Motives for compliance were the following: perceiving COVID-19 as a serious health threat, direct confrontation with critically ill COVID-19 patients, protecting someone dear from COVID-19, and being convinced of the importance of social and/or sexual distancing measures.

**Conclusions:**

Information on sexual distancing needs to be made more explicit, accessible, understandable, inclusive, customized to individual barriers, and relatable to the key populations. This may improve the effectiveness of measures and health recommendations in both the current COVID-19 pandemic and future respiratory outbreaks.

## Abstract 2

A qualitative study in a sexual health setting found that sexual distancing information to prevent severe acute respiratory syndrome coronavirus 2 transmission needs to be provided in a more explicit, accessible, understandable, and inclusive manner.

On December 31, 2019, the Wuhan Municipal Health Commission reported a cluster of pneumonia cases related to coronavirus disease 2019 (COVID-19) caused by severe acute respiratory syndrome coronavirus 2 (SARS-CoV-2).^1^ The virus rapidly spread across the world via airborne and droplet transmission. Strict measures to control COVID-19 were imposed globally. Governments instructed citizens to socially distance from persons outside their household.^2^ Social distancing includes sexual distancing, advising individuals to abstain from sexual contact with persons outside one's household.

Similar measures were imposed in the Netherlands. During the national press conference on March 15, 2020, all inhabitants of the Netherlands were instructed to keep social distance.^3^ From May 14, 2020 on, 1 sexual partner (sex buddy) outside the household was considered acceptable.^4^ Nonetheless, during this period, some of the visitors of the center for sexual health of the Amsterdam Public Health Service reported noncompliance with the social and sexual distancing measures. Research explaining noncompliance during a pandemic with potentially grave consequences is still lacking.

We aimed to elucidate the barriers and motives for social and sexual distancing (non)compliance and related factors that could have led to (non)compliance among visitors of a center for sexual health using a semistructured interview guide.

We conducted an open exploratory qualitative investigation using 2 theoretical frameworks to interpret the results. Insights in the barriers and motives for social and sexual distancing measures and related factors are required to improve communication regarding preventive measures and design interventions to curb transmission of droplet and air-borne infections in the current and in future epidemics.

## MATERIALS AND METHODS

### Study Site and Population

The center for sexual health is a specialized center for sexually transmitted infection (STI) and offers free-of-charge care for key populations, using priority criteria to get tested, for example, being younger than 25 years or a man who has sex with men (MSM), doing sex work, having STI-related symptoms, or a notification.^5^

We approached MSM visitors of the center for sexual health who were 18 years or older and did not comply (noncompliers) with sexual distancing measures during the first COVID-19 lockdown in the Netherlands (March 15–May 31, 2020). Eligibility was assessed by the health care workers of the center for sexual health at the time of the consultation. Visitors who were eligible were asked to complete the informed consent at the end of the consultation.

Noncompliers are defined as the visitors who had sex partners who lived outside one's household since the start of the social distancing measures on March 15, 2020, or had more than 1 sexual partner outside one's household since the start of the sex-buddy measure on May 14, 2020. We continued to recruit new participants until we reached thematic saturation. Since the start of the sex-buddy measure on May 14, 2020, visitors who complied with sexual distancing measures (compliers) also visited the center for sexual health. Compliers are defined as the visitors who refrained from sex with partners outside one's household since the start of the social distancing measures on March 15, 2020, or had only 1 sexual partner outside one's household since the start of the sex-buddy measure on May 14, 2020. The compliers were included as controls.

### Procedure and Analysis

Upon invitation during routine STI consultations and after obtaining written informed consent, participants were contacted by the interviewer (D.C.d.V.) and interviewed during a telephone call in English or Dutch. We asked questions on social and sexual behavior, changes in social and sexual behavior due to COVID-19, opinions toward COVID-19 and the government, and compliance to the measures (see Questionnaire, Supplement Digital Content 1, http://links.lww.com/OLQ/A818). The questions were selected through discussion by 6 researchers of the center for sexual health: D.C.d.V., H.J.C.d.V., S.D., J.L., B.J.M., K.d.J., and T.H. The interviews were transcribed verbatim by an external transcription agency. Each transcription was checked on accuracy and corrected where deemed necessary. The transcriptions were analyzed using an open-coding process in MAXQDA plus 2020 (VERBI Software, Berlin, Germany).^6^ One researcher (D.C.d.V.) coded each transcript and developed an initial coding scheme after the first 3 interviews. The scheme was revised every subsequent 3 interviews leading to the final code structure. Two other researchers (H.M.L.Z., U.D.) checked the coding scheme through discussion of variability until consensus was reached. We categorized codes into overarching codes, which formed the main themes and reflect the barriers and motives for sexual distancing and related factors that could have led to (non)compliance. We asked participants to score satisfaction with their sex life before and during the lockdown using a scale from 1 (extremely dissatisfied) to 10 (extremely satisfied).

#### Theoretical Framework

To interpret the results, we used 2 different psychological models previously used to explain barriers and motives in HIV prevention measures: the information–motivation–behavioral skills (IMB) model^7^ and the health belief model (HBM).^8,9^

The IMB model states that information and motivation both have a direct effect on behavior and are able to activate behavioral skills resulting in the initiation and maintenance of specific health behavior (see the IMB model, Supplement Digital Content 2, http://links.lww.com/OLQ/A818).^7^ The HBM states that a person's engagement in health-protective behavior is explained by one's beliefs of the susceptibility and the severity of the disease, the perceived benefits or costs of engaging in the protective action, and aspects of self-efficacy (see the HBM, Supplement Digital Content 3, http://links.lww.com/OLQ/A818).^8,9^ Based on the IMB model, we expected that information regarding coronavirus transmission modes, distancing options, and the motivation to do so would have a direct effect on sexual distancing behavior. Moreover, considering COVID-19 was a newly emerging disease, we expected elements from the HBM to play an important role in establishing the motivation for sexual distancing. In particular, the perception of threat (susceptibility and severity to the coronavirus) and the gains and costs analysis for conducting the protective behavior (i.e., the gain of avoiding diseases versus the costs of abstaining from sex).

#### Ethical Considerations

The Amsterdam University Medical Centre ethics committee approved the study and deemed a full review not necessary according to the Medical Research Involving Human Subjects Act (reference letter: W20_161, No. 20.194; delivery date: April 16, 2020).

## RESULTS

### Study Population

We recruited our participants between mid-April 2020 and July 3, 2020, until thematic saturation was reached. Among 5700 STI consultations of the center for sexual health in this period, 3526 candidates were MSM and 18 years or older and eligible for interview. Of these consultations, 37 noncomplying visitors were invited by the study nurse and completed the informed consent and contact information forms. Upon consent, we approached the 37 noncomplying visitors, of which 17 noncomplying visitors did not respond to the interview invitation. Because we only wanted to include MSM, 1 female visitor was excluded from the noncompliers (incorrect invitation), and 1 recording of a noncomplying visitor proved inaudible.

Between July 8, 2020, and August 11, 2020, there were 3842 STI consultations. Among these consultations, 1923 participants were MSM and 18 years or older and eligible for interview, of which 4 complying visitors completed the informed consent and contact information forms. Upon consent, we approached the 4 complying visitors; all compliers responded to the interview invitation. We decided to stop including compliers at August 11 because, since the measures were relaxed in the summer of 2020, it became harder to define and find complying participants.

Thus, 18 noncompliers and 4 complying participants were available for the analysis (Table 1).

**TABLE 1:** Results (Created by the Authors): Characteristics of Participants Complying and Noncomplying to Social and Sexual Distancing Measures During the First COVID-19 Lockdown Period (March 15–May 31, 2020), Center for Sexual Health, Amsterdam, the Netherlands

	Noncomplying Group (n = 18)	Complying Group (n = 4)
Age, y		
20–30	2 (11%)	2 (50%)
30–40	6 (33%)	0 (0%)
40–50	7 (39%)	1 (25%)
50–60	3 (17%)	1 (25%)
Highest achieved educational attainment		
High school	1 (6%)	0 (0%)
Secondary vocational education	1 (6%)	0 (0%)
Higher vocational education	9 (50%)	3 (75%)
Academic higher education	7 (39%)	1 (25%)
Country of birth		
The Netherlands	8 (44%)	3 (75%)
Other	10 (56%)	1 (25%)
Language		
Dutch	14 (78%)	3 (75%)
Other	4 (22%)	1 (25%)
Work during COVID-19 lockdown		
Yes	13 (72%)	4 (100%)
No	4 (22%)	0 (0%)
Missing	1 (6%)	0 (0%)
Work from home		
Yes	8 (44%)	4 (100%)
No	3 (17%)	0 (0%)
Missing	7 (39%)	0 (0%)
Relationship (boyfriend/husband)		
Yes	5 (28%)	3 (75%)
No	13 (72%)	1 (25%)
Living situation		
Alone	9 (50%)	0 (0%)
Roommate	9 (50%)	4 (100%)
Partner	3 (17%)	3 (75%)
Family	1 (6%)	0 (0%)
Roommate	5 (28%)	1 (25%)
Gender of sex partners		
MSM	17 (94%)	4 (100%)
MSM + MSF	1 (6%)	0 (0%)
No. sex partners during COVID-19 lockdown		
0	0 (0%)	4 (100%)
1–5	16 (89%)	0 (0%)
6–10	1 (6%)	0 (0%)
15–20	1 (6%)	0 (0%)

MSF indicates men who have sex with females; MSM, men who have sex with men.

The duration of the interviews varied between 23 and 57 minutes. The age of the participants varied between 22 and 58 years (mean age, 40 years). Most of the participants spoke Dutch (17 of 22). Four interviews were performed in English.

### Main Themes

We identified 9 main themes: (1) perceptions about social distancing measures and the government, (2) perceived severity and susceptibility of COVID-19, (3) discrepancy between social distancing and sexual distancing, (4) sex and physical contact before and during the lockdown, (5) barriers for sexual distancing, (6) regret about not complying with sexual distancing, (7) motives for sexual distancing among compliers, (8) motives for temporary sexual distancing among noncompliers, and (9) anticipated sex life after the lockdown. Hereinafter, these main themes are discussed in more detail.

#### Perceptions About Social Distancing Measures and the Government

Some noncompliers did not understand the government information (Table 2: noncomplier 1). The widely viewed television press conferences were only in Dutch. Some stated not to have faith in the government (noncomplier 2, quote 1), or thought that the tone of the campaign and the measures were heteronormative (noncomplier 2, quote 2). This last remark was also mentioned by compliers (complier 2). Some suggested that changing the tone of the campaign could make them feel more socially understood (noncomplier 2, quote 3) and that specific changes in the measures could lead to better compliance (noncomplier 2, quote 4).

**TABLE 2:** Results (Created by the Authors): Quotes About Barriers and Motives for Sexual Distancing in Relation to Social Distancing From Participants Complying and Not Complying to Sexual Distancing Measures During the First COVID-19 Lockdown Period (March–May 2020), Center for Sexual Health, Amsterdam, the Netherlands

Barriers and Motives for Sexual Distancing in Relation to Social Distancing	
Subjects	Quotes
Perceptions about social distancing measures and the government	
NC 1, age: 47 y	“I saw the prime minister, the press conference that he did, but I didn't follow so much”
NC 2, quote 1, age: 31 y	“Sorry, I don't believe in what our government does actually…”
NC 2, quote 2, age: 31 y	“It's all about being straight, with a girlfriend, so to speak, and yes… Anything other than that is a no-go and not taken into account.”
C 2, age: 22 y	“What I also find very remarkable is the, I would almost say, ‘Christian way’ in which such a press conference is given. As if they assume that there are only households in the Netherlands. Of course, that makes no sense at all.”
NC 2, quote 3, age: 31 y	“I do think that it [changing the tone of the campaign] has some influence on feeling a bit more involved. Because now I hear a lot about families [during the press conferences], so I think it contributes to a bit of support and to being socially understood in larger terms.”
NC 2, quote 4, age: 31 y	“During such a press conference hints were made once that you had to have a regular sex buddy, mention something like this more often or give alternatives; make it workable, make it livable. Think more in terms of possibilities, instead of thinking ‘it's not allowed.’” “So, in the short term you are kind of undermining the rule by offering an alternative which is not preferable but sort of accepted or more workable, which reduces the risk of infection by 50% so to speak, but that is more than not sticking to it at all. Look then I think it [complying with the measures] will come.”
Perceived severity and susceptibility of COVID-19	
NC 3, age: 40 y	“Because I am personally not in the risk group [for a severe course of COVID-19] myself, except that I have HIV. Good immune system, I am young, haha well, quite young. I'm not worried.”
NC 4, age: 45 y	“I'm still scared, because the impact of the virus depends on the age group, but even being forty-five, there are cases of people that passed away… So, there's still a chance that, yeah, I might not be the lucky one. You never know.”
NC 1, age: 47 y	“Important [not to get infected with COVID-19], especially for work. … I can stay home. I mean, it's not a big deal, not for the company, but, you know, it would be a pity.”
NC 5, age: 39 y	“Yes, so then [when his mother was at his house] no one was allowed to come near me … What would that be? The first two months or so I really had zero-point zero contact.” “But when she was gone… you are, indeed, less precise [with complying with the measures].”
C 3, age: 58 y	“[it is very important for me not to get infected with COVID-19] because you don't know what the consequences are… Maybe it will make me very sick and it will have no consequences for me at all, but it is also an option that unpleasant lasting side effect will occur.”
Discrepancy between social distancing and sexual distancing	
NC 6, age: 31 y	“I also wonder what the actual effect has been, to limit that [sexual contact] very much. I think it is more important to avoid major outbreaks of contamination, like big events inside bars and things like that. And I also suspect that the ‘one-on-one contact’ transmission will be quite limited.”
NC 7, age: 58 y	“I basically completely isolated myself for one month. I was not even going outside for groceries, nothing. I was ordering online.” [while being a noncomplier of sexual distancing]
Sex and physical contact before and during the lockdown	
NC 8, quote 1, age: 45 y	“It [sex during COVID-19] has mainly declined in quantity… I don't meet new, different guys. And I also have a slightly different meaning with sex. It should have a little more substance in the term of friendship.”
NC 9, age: 30 y	“Yes, the urge for sex has changed. … It fluctuates. So, it started that I immediately thought, also a bit out of fear of corona, that I thought, “Oh, my libido is very low.” No need for it [sex] at all.”
NC 4, age: 45 y	“Yeah, actually, this is because I think that I'm a little bit more busy at work and my stress levels are higher, so I feel less… Yeah, I'm less playful, let's put it this way.”
NC 10, age: 53 y	“I think just human contact [is what is missed]. Everything is just a bit more distant. And yes, I have some difficulties with that distance, yes. In general.”
NC 2, age: 31 y	“[The way of contacting potential sex partners during the pandemic is] Much more online actually…” “Before [COVID-19] also apps, parties, but mostly parties”
NC 8, quote 2, age: 45 y	“For example, I got Grindr off my phone … I'm not meeting new, different guys.”
NC 11, age: 50 y	“So that whole list of ‘have you had any COVID-19 risk lately?’ and ‘did you have any human interactions at all?’ [was asked before meeting up] Yes, plus of course the question if he had any symptoms in the recent weeks.”
NC 12, age: 39 y	“We don't kiss anymore or… Well, I don't perform fellatio.”
C 2, age: 22 y	“Well ‘missing’ [sex] is a bit strong, but well it [sex] is fun to do, but it didn't drove me crazy that it wasn't possible. I was at peace with it. I didn't think that it [abstaining from sex outside the household] was a big deal.”
C 1, quote 1, age: 49 y	“I notice that by focusing on other things; work, the upcoming renovation, family and friends, that the sex drive decreases.”
C 1, quote 2, age: 49 y	“Hugging people you know and friends and giving them a kiss. I miss that even more than just the sex.”
Barriers for sexual distancing	
NC 12, quote 1, age: 39 y	“If I was aware of that [sexual distancing being part of social distancing], I would have probably just minimized it [sexual contact]”.
NC 13, age: 43 y	“But if you are single, it [not having sex] is a bit difficult over a long period.”
NC 12, quote 2, age: 39 y	“When it first started, it was hard, because, I mean, I really do have a lot of sex.”
NC 6, age: 31 y	“Well, it was actually more the social aspect [what is missed], actually. Just say, the bit of attention, the bit getting to know someone. Because it was a somewhat lonelier period anyway. That also played a part in that [having sexual contact despite the measures].”
NC 1, age: 47 y	“Putting my pleasure… or wanting pleasure more than the real danger that it had.”
NC 14, age: 49 y	“And I think there was also a little bit of alcohol and a little bit of ecstasy involved. As a result, my inhibitions somewhat faded.”
NC 3, quote 1, age: 40 y	“When you hear that 60 to 70% of the population is going to get it [COVID-19], I think: I'm not going to wait a year and a half for the vaccine to not have sex.”
NC 7, age: 58 y	“Because I saw that the curve was flattening. There were less cases and also nobody was traveling anymore, So… Yes, I thought: “Okay, now I can do it again.”
NC 15, age: 27 y	“And, I think, maybe it was also a factor that you are less afraid yourself of getting corona than before. In the beginning we were still a bit panicked. Yeah, it's also a bit… You, maybe kind of become immune to all the warnings and corona panic.”
NC 3, quote 2, age: 40 y	“One thing that I noticed… I've been kind of stressed out about because of what happened to my company, my profession… I noticed that sex itself is just a coping mechanism for me. Especially in the beginning with so much stress, so much… you know? Wanting to bury my head in the sand and then sex is a kind of an escape.”
NC 10, age: 53 y	“I work as a **” - Interviewer: And do you have any other activities, besides the job, that you must leave the house for? “Yes, I do sex work” - Interviewer: So, you do the sex work as an addition to your job as a ** “Yes, I happen to have a job again; I always have a job now.” - Interviewer: Is sex work something to bridge the time between jobs? “Yes, it was, but it really isn't anymore” - Interviewer: can I say that you continue because enjoy doing it now? “Yes”
Regret about not complying with sexual distancing	
NC 11, age: 50 y	“Yes, I already failed once… [when having sexual contact despite the measures]”
NC 9, age: 30 y	“Yes, I felt quite guilty about it afterwards. Now I have not helped very well with the constraining of that first big wave.”
Motives for sexual distancing among compliers	
C 1, quote 1, age: 49 y	“My husband has been very ill … and all signals pointed at Corona at the time. All tests showed it was not Corona, but it had all the signs of it [COVID-19] and he stayed in the Corona department. We saw very closely how serious that was. He was quite ill. And three days of oxygen and things like that. We have seen then that we have to take that [COVID-19] seriously, yes. We've seen that all up close.”
C 2, quote 1, age: 22 y	“I found the news reports that we had from Italy at one point…, I found that much more disturbing; those balloons around those heads and also those young people of seventeen and stuff, I thought that was very intense and I was more put off by that than by Mark [Rutte, Dutch prime minister] who said that everyone would get it.”
C 2, quote 2, age: 22 y	“I thought it was more important that I could see the people around me than that I would take, say, risks of Corona out there with a stranger.”
C 1, quote 2, age: 49 y	“And I think we also look at it from the idea of… If we look at friends and relatives, people who are alone, people who don't have their jobs anymore, then the two of us consider ourselves very lucky that we can just continue. Maybe also a bit from the attitude of: “Well, that one and a half meters distance, we have to be able to stick to that constantly, it seems to me, in this very good position that we have.”
Motives for temporary sexual distancing among noncompliers	
NC 9, age: 30 y	“So it started that I immediately thought, also a bit out of fear of corona, that I thought: ‘Oh, my libido is very low, no needs at all.’ And at some point, it will come back and that's when I had sex again for the first time….” “It comes back automatically because it has been taking so long.”
NC 16, age: 37 y	“I am a Muslim, and now that it is Ramadan, you are actually not allowed to do anything [sex related] and I have not done anything.”
Anticipated sex life after the lockdown	
NC 9, age: 30 y	“I expect that my sex life will become in any case more frequent and more active.”
NC 8, age: 45 y	“Because yes, I have been hunting through the city with a lot of sexual desire for several years, and actually I think my life is quite okay the way it goes [during the pandemic]”.

C indicates complier; NC, noncomplier.

Other critical remarks on the lockdown measures were its unclear communication, lack of substantiation, impracticality, and exaggeration.

#### Perceived Severity and Susceptibility of COVID-19

Being young and healthy, some noncompliers considered themselves not at risk for severe illness (noncomplier 3). However, others feared the COVID-19–related morbidity and mortality (noncomplier 4), consequences such as being unable to go to work (noncomplier 1), or the risk to infect their loved ones (noncomplier 5). Both noncompliers and compliers (complier 3) mentioned fear for COVID-19, although this did not lead to compliance in the first group.

#### Discrepancy Between Social Distancing and Sexual Distancing

Some noncompliers doubted the effectiveness of sexual distancing and thought that it was more important to avoid large venue gatherings (noncomplier 6). Where some did not comply with sexual distancing, they did comply with social distancing measures such as not seeing their family or completely isolating themselves (noncomplier 7). This discrepancy was not seen among compliers, all of whom complied with both sexual and social distancing.

#### Sex and Physical Contact Before and During the Lockdown

Most participants adjusted their sex life; for example, they had fewer anonymous sex partners and/or fewer sex partners in general (noncomplier 8, quote 1). Some experienced less need for physical and sexual contact, either out of fear for COVID-19 (noncomplier 9) or because of an increased workload (noncomplier 4). Others had an increased desire for sexual and/or physical contact (noncomplier 10). Some participants changed the way they sought sex partners. With the closure of bars and clubs, only dating apps and cruising areas remained as options (noncomplier 2). Some discontinued using dating apps or cruising, because they did not want to meet new partners (noncomplier 8, quote 2). Some inquired if their sex partner had COVID-19–related symptoms before having sex (noncomplier 11), whereas others avoided kissing or fellatio (noncomplier 12).

Some compliers mentioned not feeling that much sexual desire (complier 2) or managing sexual desire by distracting themselves (complier 1, quote 1). Some also mentioned that they missed physical contact more than sexual contact (complier 1, quote 2).

All participants scored their sex life lower during the lockdown period compared with before: the noncomplying group with 5.7 and 7.9 and the complying group with 3.3 and 8.0, respectively.

#### Barriers for Sexual Distancing

Reasons mentioned for noncompliance were the following: not being aware of the imposed sexual distancing measures (noncomplier 12, quote 1), difficulties refraining from all sex for the people who do not have a live-in sexual partner or are single (noncomplier 13), being used to a very active sex life before the lockdown (noncomplier 12, quote 2), missing the social aspect of having sex (noncomplier 6), a very strong urge for sex (noncomplier 1), and being under the influence of alcohol and/or drugs (noncomplier 14).

Early in the pandemic, the prime minister mentioned that most of the population would become infected before group immunity would dampen the pandemic. One participant concluded that COVID-19 infection was thus inevitable and therefore not willing to adhere (noncomplier 3, quote 1). Some gave up sexual distancing when the number of infections declined rapidly toward the end of the first lockdown (noncomplier 7), or when they got tired with “COVID-19 panic,” referring to the constant alarming media reports (noncomplier 15). Some noncompliers mentioned sex as a coping mechanism to deal with the pandemic impact, like losing their job or financial concerns (noncomplier 3, quote 2). One participant continued his sex work during the lockdown for fun, even though he did not need the financial benefits (noncomplier 10).

#### Regret About Not Complying With Sexual Distancing

Some participants spontaneously mentioned regret for noncompliance with sexual distancing measures. One felt as if he had failed (noncomplier 11), and another felt sorry for potentially contributing to SARS-CoV-2 transmission (noncomplier 9).

#### Motives for Sexual Distancing Among Compliers

Motives to comply with sexual distancing were the following: being scared of infection because of a direct confrontation with the impact of COVID-19 (complier 1, quote 1) or media reports on seriously ill COVID-19 patients (complier 2, quote 1), not wanting to endanger family and friends (complier 2, quote 2), or being satisfied with the current living situation including having a partner within the household (complier 1, quote 2).

#### Motives for Temporary Sexual Distancing Among Noncompliers

Some noncompliers complied with the sexual distancing measures in the beginning of the lockdown but failed later on when their sexual desire increased (noncomplier 9). Others complied for religious reasons only, for example during the Ramadan period (noncomplier 16).

#### Anticipated Sex Life After the Lockdown

After the lockdown, some noncompliers expected to have more sex partners and more sex (noncomplier 9), whereas others expected to continue their sex life during lockdown also after social distancing measures are lifted because they enjoyed their sex life better than before (enjoying not constantly looking for sex, noncomplier 8).

## DISCUSSION

Here we describe the barriers and motives for social and sexual distancing and related factors during the first COVID-19 lockdown in the Netherlands using a semistructured interview guide. Our main findings are the following: (1) as a barrier, some participants did not understand the government information, (2) as a barrier or motive, participants developed a personal COVID-19 risk assessment in which they weighed their personal risk of infection and/or severity of illness and the risk to infect their social contacts against the benefits that would result from sexual contact, and (3) as a barrier or motive, participants changed their risk assessment during the lockdown based on new information or changes in their needs or desires.

Sexual distancing is part of the broader social distancing concept; however, studies disentangling these 2 related preventive measures are lacking. Information about how people who are sexually active understand and cope with these strategies can help in improving communication and adherence to such measures. To the best of our knowledge, qualitative studies evaluating the concordance between social and sexual distancing have not been performed yet. We decided to use “ad verbatim” English translations of the quotes of the Dutch participants as much as possible. As a result, some might sound awkward. Only when this resulted in ambiguity, the quotes were modified for clarification by D.C.d.V.

(1) A strong point of our study is its timely character. We started the study early in the COVID-19 pandemic and interviewed the first participant when the measures had been imposed for just 2 months. At this moment, vaccinations were not available, so it was unsure when the vaccination campaign would start. The vaccination campaign in the Netherlands started on January 8, 2021 (8 months after our study took place). (2) We approached both participants who complied and who did not comply with the sexual distancing measures, to identify distinguishing factors and get a full overview of both barriers and motives for (non)complying to the measures. (3) Because we recruited in an early phase of the pandemic, at the time, not many alternative and conspiracy theories were circulating yet, although some participants did mention a lack of trust in the government.^10^

Our study has some limitations. (1) The complying group only consisted of four participants. To avoid heterogeneity, we discontinued to include participants in this group after the sexual distancing measures were loosened and a single sexual contact outside the household (sex buddy) was allowed. This may have led to a less comprehensive overview of motives to comply with sexual distancing measures. (2) Because we only approached MSM visiting the center for sexual health in Amsterdam, the outcome cannot be generalized to other communities.

Some of our findings were supported by a recent quantitative study on sexual behavior among MSM during COVID-19 restrictions in Amsterdam.^11^ Having had an active sex life before COVID-19 as a barrier for compliance was mentioned both in the study by van Bilsen et al.^11^ and our study. Barriers/motives found here, but not studied by van Bilsen et al., were the lack of information on the need for sexual distancing, heteronormativity of the measures/communication, wanting to protect someone dear, and being scared by media reports. Furthermore, we found that some noncompliers adhered to social but not to sexual distancing measures.

Bowling et al.^12^ examined the risk perceptions related to sexuality during the COVID-19 pandemic and found that (in line with our results) risk perceptions around sex now included COVID-19–related risks. Walsh et al.^13^ and Jongen et al.^14^ examined behavior changes and sexual agreement changes in MSM during the COVID-19 pandemic. Similar to our study, they both found a decrease in the number of casual sex partners and an increase in monogamy during the pandemic. These shifts in partner type, however, returned to the prerestriction levels once the measures were lifted. Craig-Kuhn et al.^15^ studied changes in sexual behavior in heterosexual males during COVID-19 stay-at-home orders. They also found a reduction in sexual frequency and that only 27.9% had seen information about safe sex during the pandemic, indicating that the information provision on sexual distancing during the pandemic should be emphasized. Holloway et al.^16^ examined the effect of sexual distancing on MSM. Similar to our study, they found a decrease in satisfaction with their sex life during the distancing measures. Our qualitative results can be used to design future quantitative studies on the impact, relevance and magnitude of sexual distancing as a preventive measure in air-borne infections.

We used the IMB^7^ and the HBM model^8,9^ to interpret our results. Although most participants realized that sexual distancing was an obvious part of social distancing, not all were aware of the need for sexual distancing to prevent transmission because of language barriers in non-Dutch speakers and a lack of emphasis on sexual distancing in the communication during the lockdown. This is in line with the IMB model that states that information is key in the compliance to preventive measures. The motivation component of the IMB model explained why some participants did not comply with the distancing measures. For instance, the group immunity quote from the prime minister directly dampened the motivation in one of our participants. Other barriers leading to noncompliance were a tradeoff between the benefits of having sex and a risk perception of COVID-19. This is also in line with the HBM, which states that people's perceived disease susceptibility and severity correlate with the engagement in health-promoting behavior.^17^ The behavioral skills component of the IMB model concerns the objective ability to perform health behavior and the self-efficacy for this behavior.^18^ Using sex as a coping mechanism to deal with the negative impact of the pandemic and being used to a prepandemic active sex life are examples of behavioral barriers found in our study.

In accordance with the HBM, the perceived severity of COVID-19 and the benefits of sex played an important role in compliance. Participants complied if the perceived severity of COVID-19 outweighed the perceived sexual benefit. Some participants were able to comply with sexual distancing at first, but the fear of getting infected with SARS-CoV-2 subsided in time and the urge for sex and physical contact took over, contributing to noncompliance later on. Media messages also have an important role in the motivation for sexual distancing. The perceived inevitability to get infected in the long run and the expected long duration before a vaccine would be available had a negative impact on sexual distancing compliance. Protecting someone dear from getting infected, on the other hand, had a positive effect on sexual distancing compliance.

Information is an important factor to initiate specific behavior. Government health information could be improved and be made more accessible and understandable, also for those not fluent in the native language. Moreover, the role of sexual contact in the transmission of SARS-CoV-2 seemed not to be self-evident in this study. In the first government press conferences, the ban on sex work was the only measure referring to the potential sexual transmission risk.^19^

Some participants found the health information heteronormative and not inclusive. Providing information actively targeting minority populations (LHBTI+, migrants) might be needed. This might also apply to Dutch citizens from Turkish or Moroccan descent who primarily received their information via media channels from their country of origin.^20^ This information sometimes conflicted with Dutch health measures leading to confusion and noncompliance.

One of the complying participants feared getting infected with COVID-19 when media showed images of young people of his age who were admitted to intensive care units. This motivated him to comply with sexual distancing. Likewise, the perceived risk of HIV is one of the motives to use preexposure prophylaxis.^21,22^

Some of the participants in our study population decided to only have sex with partners they already knew before the COVID-19 pandemic started. The idea that “known partners are safe partners” is a phenomenon widely practiced in an attempt to reduce HIV acquisition (serosorting).^23,24^

Our findings suggest that the government health information on sexual distancing to prevent the transmission of SARS-COV-2, and future droplet and airborne pathogens needs to be made more explicit, accessible, understandable, inclusive, and relatable to key populations. This can improve effective measures against droplet and air-borne infections in the current and in future epidemics.
