# Supplementary material for: Barriers and Motives for Complying With “Sexual Distancing” Among Men Who Have Sex With Men During the First COVID-19 Pandemic Lockdown in Amsterdam: A Qualitative Study
Source: Sex Transm Dis. 2022 Apr 9;49(7):497–503. doi: 10.1097/OLQ.0000000000001636 (PMC9196919; doi:10.1097/OLQ.0000000000001636)
Supplement: SUPPLEMENTARY MATERIAL [file std-49-497-s002.docx]

Supplement References

31S. Heathcote EJ, Marcellin P, Buti M, et al. Three-year efficacy and safety of tenofovir disoproxil fumarate treatment for chronic hepatitis B. Gastroenterology 2011;140:132-43.

32S. Lok AS, McMahon BJ, Brown RS, Jr., et al. Antiviral therapy for chronic hepatitis B viral infection in adults: A systematic review and meta-analysis. Hepatology 2016;63:284-306.

33S. Nguyen MH, Yang HI, Le A, et al. Reduced Incidence of Hepatocellular Carcinoma in Cirrhotic and Noncirrhotic Patients With Chronic Hepatitis B Treated With Tenofovir-A Propensity Score-Matched Study. J Infect Dis 2019;219:10-18.

34S. Papatheodoridis GV, Chan HL, Hansen BE, et al. Risk of hepatocellular carcinoma in chronic hepatitis B: assessment and modification with current antiviral therapy. J Hepatol 2015;62:956-67.

35S. Tenney DJ, Rose RE, Baldick CJ, et al. Long-term monitoring shows hepatitis B virus resistance to entecavir in nucleoside-naive patients is rare through 5 years of therapy. Hepatology 2009;49:1503-14.

36S. Wong GL, Chan HL, Mak CH, et al. Entecavir treatment reduces hepatic events and deaths in chronic hepatitis B patients with liver cirrhosis. Hepatology 2013.

37S. Arias E. United States Life Tables, 2017. Natl Vital Stat Rep 2019;68:1-66.

38S. Services CfMM. Physician Fee Schedule

39S. Centers for Disease Control and Prevention. CDC Vaccine Price List. <https://www.cdc.gov/vaccines/programs/vfc/awardees/vaccine-management/price-list/index.html#adult>. Volume 2021, 2021.

40S. Entecavir and Tenofovir. In: RedBook. Green Village (CO): IBM Corporation; 2021 [cited 2021 Jan 22]. Available from: <www.micromedexsolutions.com>. Subscription required to view.

41S. Harris AM, Osinubi A, Nelson NP, et al. The Hepatitis B Care Cascade Using Administrative Claims Data, 2016. Am J Manag Care 2020;26:331-338.

42S. Liu S, Cipriano LE, Holodniy M, et al. New protease inhibitors for the treatment of chronic hepatitis C: a cost-effectiveness analysis. Ann Intern Med 2012;156:279-90.

43S. Statistics BoL. CPI Inflation Calculator. Volume 2016.

44S. Terrault NA, Bzowej NH, Chang KM, et al. AASLD guidelines for treatment of chronic hepatitis B. Hepatology 2016;63:261-83.

45S. Woo G, Tomlinson G, Yim C, et al. Health state utilities and quality of life in patients with hepatitis B. Can J Gastroenterol 2012;26:445-51.

46S. Toy M, Hutton DW, So S. Population Health And Economic Impacts Of Reaching Chronic Hepatitis B Diagnosis And Treatment Targets In The US. Health Aff (Millwood) 2018;37:1033-1040.

47S. Toy M, Hutton D, Harris AM, et al. Cost-Effectiveness of 1-Time Universal Screening for Chronic Hepatitis B Infection in Adults in the United States. Clin Infect Dis 2022;74:210-217.

48S. Trepka MJ, Weisbord JS, Zhang G, et al. Hepatitis B virus infection risk factors and immunity among sexually transmitted disease clinic clients. Sex Transm Dis 2003;30:914-8.

49S. Centers for Disease Control and Prevention. CDC vaccine price list.

50S. Tsai Y, Zhou F, Lindley MC. Insurance Reimbursements for Routinely Recommended Adult Vaccines in the Private Sector. American journal of preventive medicine 2019;57:180-190.

51S. Ray KN, Chari AV, Engberg J, et al. Opportunity costs of ambulatory medical care in the United States. Am J Manag Care 2015;21:567-74.

52S. Prosser LA, O'Brien MA, Molinari NA, et al. Non-traditional settings for influenza vaccination of adults: costs and cost effectiveness. Pharmacoeconomics 2008;26:163-78.

53S. Centers for Disease Control and Prevention FDA aUDoHaHS. National Ambutarory Medical Care Survey: 2013 State and National Summary Tables. National Center for Health Statistics., 2013.

54S. Statistics BoL. <https://www.bls.gov/news.release/empsit.t19.htm> Volume 2021.

55S. Federico CA, Hsu PC, Krajden M, et al. Patient time costs and out‐of‐pocket costs in hepatitis C. Liver International 2012;32:815-825.

56S. Levie K, Gjorup I, Skinhøj P, et al. A 2-dose regimen of a recombinant hepatitis B vaccine with the immune stimulant AS04 compared with the standard 3-dose regimen of Engerix-B in healthy young adults. Scandinavian journal of infectious diseases 2002;34:610-614.

57S. Treadwell TL, Keeffe EB, Lake J, et al. Immunogenicity of two recombinant hepatitis B vaccines in older individuals. The American journal of medicine 1993;95:584-588.

58S. Joines RW, Blatter M, Abraham B, et al. A prospective, randomized, comparative US trial of a combination hepatitis A and B vaccine (Twinrix®) with corresponding monovalent vaccines (Havrix® and Engerix-B®) in adults. Vaccine 2001;19:4710-4719.

59S. Hirst A, Hyer RN, Janssen RS. Comparative cost-effectiveness of a 2-dose versus 3-dose vaccine for hepatitis B prevention in selected adult populations. Vaccine 2021.

60S. Reagan-Steiner S, Yankey D, Jeyarajah J, et al. National, Regional, State, and Selected Local Area Vaccination Coverage Among Adolescents Aged 13-17 Years--United States, 2014. MMWR Morb Mortal Wkly Rep 2015;64:784-92.
